# Co-creation of the Digital Democracy and Data Commons Manifesto: alternative sociotechnical visions of data

**DOI:** 10.12688/openreseurope.17020.2

**Published:** 2024-10-25

**Authors:** Enric Senabre Hidalgo, Antonio Calleja, Ricard Espelt, Sara Suárez Gonzalo, Mayo Fuster Morell, Andreu Belsunces

**Affiliations:** 1 Universitat Oberta de Catalunya, Barcelona, Catalonia, Spain

**Keywords:** Data Commons, Digital Democracy, Co-creation, Participatory Design, Sociotechnical Systems.

## Abstract

Amid public concern surrounding the proprietary and exploitative use of personal data by corporations and public institutions, and its consequences from a sociotechnical perspective, narratives around digital commons have recently emerged, framing potential alternatives. This paper presents the co-creation of the Digital Democracy and Data Commons Manifesto through a collaborative writing sprint, drawing on principles of openness, diversity, and inclusivity. The manifesto articulates a technopolitical vision for data governance that prioritizes community control over data. We analyze the manifesto's evolution throughout the process, demonstrating its capacity to address contemporary concerns such as data extractivism and algorithmic governance. Our approach is based on participatory design methods, more concretely on a collaborative writing sprint, to co-create a manifesto on alternatives to current datafication, digital inequalities, and lack of citizen control over personal data. On the one hand, we describe the process of implementing a sprint approach for collaboratively writing a topic-specific manifesto, in the context of the broader EU project DECODE (Decentralised Citizen Owned Data Ecosystems). On the other hand, we present and analyse the main results from the content structure of the manifesto over its initial and final versions, which moved progressively as a cohesive text away from a scholarly and policy-oriented tone.

## Introduction

Growing public debate is taking place around personal data protection in the digital society. More and more aspects of people's lives are mediated by digital media channels, and a myriad of platforms is exploiting huge quantities of data from billions of users ( Figueiras, 2021). Corporations and states gather and process them with the aim of surveilling and interfering with people's lives, usually for profit and/or with social control purposes. This emerging socio-economic paradigm has been variously named data-capitalism ( West, 2019), platform-capitalism ( Srnicek, 2017) or surveillance-capitalism ( Zuboff, 2019) and has provoked increasing criticism. In order to better understand and contribute to alternative visions regarding such debate, as well as to advance towards a real digital public sphere and the digital commons ( Fuchs, 2021), defying these frameworks from a sociotechnical perspective is needed. From an actor-network theory perspective ( Callon, 1987; Latour, 2007), sociotechnical systems ( Callon, 2004) involve social and technological actors, factors and dynamics. Thereby, these systems can be seen as co-produced or composed by different realities that influence their stabilization and transformation ( Miller & Jasanoff, 2004): regulations ( Borrás & Edler, 2020), infrastructures ( Bowker, 1996), narratives ( Goffman, 1974; Scheufele, 1999), social imaginaries ( Taylor, 2004) and expectations ( Wilkie & Michael, 2009), all taking part in the construction of the sociotechnical. In recent years, discussions on data commons have grown, encompassing various aspects of data governance, privacy, and public value ( Fuchs, 2021; Ostrom, 1990; Purtova & van Maanen, 2023). These works outline how commons-based approaches can provide alternatives to proprietary models of data control, aligning with the growing call for more democratic and community-oriented frameworks in the digital realm.This article challenges the prevailing techno-determinism by proposing a participatory model that integrates narratives, infrastructures, and shared expectations, essential for collective governance. We argue that this theoretical approach, which combines storytelling with socio-political dynamics, is a necessary framework for practical interventions in data governance. This article challenges the dominant paradigms in data governance by proposing a more participatory model. As Micheli *et al.* (2020) and Fischli & Muldoon (2024) argue, data governance requires not only technical solutions but also governance frameworks that promote collective control and democratic participation.

In this sense, the concept of sociotechnical imaginaries ( Jasanoff & Kim, 2015), defined as “collectively held, institutionally stabilized and publicly performed visions of desirable futures, animated by shared understandings of forms of social life and social order attainable through, and supportive of, advances in science and technology”, seems key for understanding how the symbolic layer of “data” is perceived, exploited, performed, sustained, and challenged by various actors -such as corporations, governments, think tanks, activists and communities of practice. This is also true for sociotechnical systems, considering how narratives and imaginaries are co-constructed within the social (e.g. communities, practices) and the technological (e.g. devices, knowledge), as two realities that are inextricably entangled. This implies, therefore, that transforming sociotechnical systems requires the articulation of alternative narratives and imaginaries.

For such articulation, building alternative forms of dealing with data can benefit from co-developing alternative sociotechnical systems and contributing to transforming the processes and practices by which they are built. While the process aimed for inclusive participation, expert stakeholders from the DECODE consortium played a central role in shaping key aspects of the manifesto. Their influence was particularly evident during the initial debates, where technical frameworks were established, which later shaped the manifesto's structure. However, every effort was made to ensure that the collective process allowed all voices to contribute equally. This can be done either by following the traditional “technocentric” and “technocratic” approach (in which technology is the central object of attention, and experts primarily shape the system), or from a more “sociocentric” and “sociocratic” angle (where a variety of actors shape technologies through a variety of negotiations), as sometimes described by STS scholars ( Douglas, 2012). For the latter, a valid approach is to enable participatory and deliberative models to transform sociotechnical systems promoting social transformation, as an opportunity for citizen re-appropriation and governance of data commons, without exclusive intellectual property ( De Rosnay & Musiani, 2020). Trying to avoid techno-centrism, and applying narrative and participatory techniques to a complex topic such as data, can allow us to experiment with multiple and interlinked facets, while seeking to galvanize communities around such narratives. In other words, aligning narratives and communities as the base for sociotechnical transformations which are not, and cannot be, merely “techno-centric”.

### The DDDC pilot

**Figure 1.**
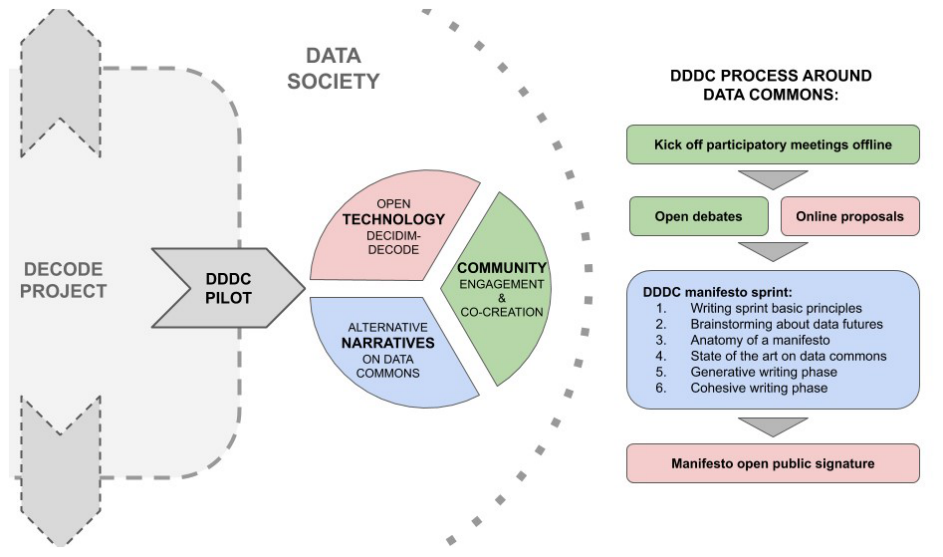
Overall process of the manifesto sprint in relation to DDDC Pilot and the DECODE project. Own elaboration.

This socio-centric approach was a key aspect of the Digital Democracy and Data Commons (DDDC) pilot, a 6 month-long participatory process embedded within the EU project DECODE. DECODE (acronym for Decentralized Citizen Owned Data Ecosystems) was a three-year Horizon 2020 EU project oriented to build new technological, legal, economic and narrative tools to rebuild our relation to data, moving it from the current forms of data extractivism and surveillance towards a model defined by data sovereignty and data commons. A model in which people could have more personal and collective control and get more benefit from the data they generate in their digital life. In this context, the DDDC (Digital Democracy and Data Commons) pilot was a 6-month process run both in Barcelona and online through several digital strategies and 10 face to face meetings. The process articulated three elements: (1) open technologies based on the open source software Decidim –a digital platform for participatory democracy, used by more than 1 million people and 450 organizations worldwide, ranging from states to cities and from activists to economic organizations ( Roth *et al.*, 2019)–; (2) community-focused co-creation processes and engagement activities around such digital infrastructure, online and offline (in Barcelona); and (3) the development of alternative narratives around the concept and practices of data commons. Within the DDDC process, the co-creation took a series of steps: kick off, face to face debates, online proposals, and, crucially, a DDDC “manifesto sprint” whose main aim was to develop a collective discussion and writing process for creating a concrete, shared narrative about data commons ( Figure 1).

This paper presents and analyzes the results of the collaborative process for co-creating the “Digital Democracy and Data Commons Manifesto” ( DDDC Manifesto). The process instantiated the key notion behind our approach: the practice of co-creating critical sociotechnical narratives. As a key output of the mentioned pilot, the manifesto was based on the techno-political and democratic purview of the Decidim and DECODE projects. Unlike the previous tradition of manifestos, the DDDC Manifesto was not the product of an individual reflection or authorship, nor was it only focused on socio-political claims; it was the result of an open and collaborative process of writing and deliberation. The participants in this co-creation process ranged from tech-enthusiasts to data commons advocates, each bringing diverse perspectives on data governance. While some had concerns about corporate data practices, others were already aligned with commons-based approaches. This diversity influenced the direction of our discussions, as we sought to challenge both complacency and corporate influence in data governance.

Our aim is to describe and analyze the overall process of the DDDC Manifesto co-creation, contributing to the study but also development of sociotechnical narratives in current debates and fast-paced disruptions around data extractivism and data sovereignty. The paper is structured into six sections. The second one (after this Introduction) delves into the theory of manifestos and the role of deliberation and participatory processes in their creation, as well as on the relationship of these topics to the theory of commons and commoning. Section three explains how and why the manifesto was conceived as part of the DDDC Pilot. It also specifies the methodology followed for the creation of the manifesto. Results of the whole process are presented and discussed in section four, additionally relating the described findings to previous studies and literature in section five. Conclusions, in section six, include account of the limitations of our case-study methodology and evaluative approach, as well as a discussion of further research needed.

## Co-creating visions: manifestos and design methodologies

In modern times, a paradigmatic channel for articulating alternative narratives and visions of society was the manifesto. From the *Communist Manifesto* in the nineteenth century to the *Accelerationist Manifesto* in the twenty-first, they have been drafted to synthesize ideas, articulate demands, mobilize human collectives and embody social visions. These two paradigmatic, well-known examples tended to focus on markedly social or economic claims. The *Communist Manifesto* referred to technology more as a ready-made tool for economic and social emancipation, while the *Accelerationist* one saw technology more as a space to be constructed, shaped and inhabited according to political ideals and material practices. Manifestos themselves were understood, in this sense, as “means” rather than “mediations” (as *intermediaries* instead of *mediators*, in Latour's, 2007 terms): only as a tool created by a few authors in order to convince others, rather than a process in which both the authors' and the audiences' visions emerged concurrently.

From well-known examples in the political and artistic domains in the XIX and early XX centuries, to more recent examples involving digital technologies, such as the *Cyborg Manifesto* in the 80s, the persuasive features of manifestos are usually counterposed to traditional academic language ( Ashby *et al.*, 2017). However, manifestos have been considered a performative textual and narrative format ( Hanna, 2019) with potential to bridge scholarly or expert knowledge with wider audiences, and they have also been noted as civic engagement strategy tools ( Freedman, 2018). Usually in relation to the public sphere, manifestos have been studied as modern discursive forms with extremely plural and open formats, as narrative artefacts expressing and reacting to sociopolitical changes and uncertainties. From ”classical” pre-war avant-garde manifestos, to post-WWII counterculture and cyberfeminist ones, the genre has evolved as a sort of transdisciplinary writing practice with a persuasive, transformational style. They are programmatic texts that invite action without proposing a closed political program, and where knowledge is usually asserted rather than developed ( Yanoshevsky, 2009). However, due to its multiformity, there's no clear agreement on which narrative forms should strictly be considered as manifestos, when as defined by Lyon (1999) “each manifesto is context dependent, composed under historical and geographical specificities, differing both from other kinds of documents, and from other manifestos”.

Due to a recent proliferation of digital and technology related manifestos in Europe, and more concretely in the field of Human Computer Interaction, it’s currently possible to find new perspectives within this genre that incorporate critical reflective attention to the impacts, values and challenges of digitalization, like for example in the field of the Internet of Things ( Fritsch *et al.*, 2018). Their intended function as frameworks of directions to improve the practice of digital design and development, and for more ethical approaches to technology, tend to point to questions such as transparency, openness, justice and responsibility. They move from the descriptive to the prescriptive and even the prospective, working rhetorically, “between what has been done and what will be done, between the accomplished and the potential” ( Caws, 2001).

In order to consider the relationship between the genre of manifestos and recent advances in the theory and practice of the Commons, we must start from how these operate as cultural and symbolic resources that can create new avenues for collective action ( Sandström *et al.*, 2017), also activating imaginaries that can build a sense of community based on mutuality and rationality ( Harcourt, 2019). It is from this perspective that we can approach and understand recent manifestos around the concept of digital commons that start to incorporate, albeit timidly, mentions to the usual extractivism of large platforms capturing the value of social exchange by monetizing the data and selling user’s 'attention' to advertisers ( Bauwens *et al.*, 2019). Or manifestos that, starting from feminist and transition perspectives, address the importance of the commons as a shared culture, prior to the structuring of alternatives ( Troncoso & Utratel, 2019), as well as the key concept of public service around digital commons ( Fuchs & Unterberger, 2021). In this sense, as indicated by De Rosnay and Stalder (2020) “the constitution of data commons also needs to overcome the apparent contradiction between personal data and property, and between privacy and open access, as a personal data commons would not lead to sharing personal information, but to govern their reuse according to values of the digital commons”.

The DDDC Manifesto involved the idea of technopolitical democratization ( Calleja-López, 2018) that characterizes the Decidim project. In that sense, it situated participation and deliberation at its core. Participation was understood as a form of “taking part as peers” ( Roth *et al.*, 2019) and thereby as a commitment to openness and inclusion of anyone and everyone ( Ranciere, 2005) on equal terms (rather than as a function of knowledge, expertise, money or credentials), and in the tradition of participatory democracy ( Barber, 2003). This approach to deliberation, in turn, implied a commitment to the exchange of publicly accessible and reasoned proposals, views, and judgments, oriented to reach agreements open to amendment in the future (to put it with Gutmann & Thompson, 2004), in a process in which the only acceptable force is the force of the better argument, as reclaimed by Habermas. Crucially, though, nurturing inclusion on the deliberative front implied exploring other forms of reasoning, interacting and world-making beyond the logo-centric and passionless modes typically associated with the deliberative tradition. As we show below, alternative methodologies of deliberation involving speculation, imagination and play were deployed during the co-creation of the DDDC manifesto.

### The data commons manifesto within the DDDC pilot

In particular, the DDDC Manifesto follows a systematic co-creation approach ( Senabre Hidalgo *et al.*, 2022). The pilot had the key goal of enriching and testing a technological architecture developed to give users more control over their data. In the terminology of the project, it would increase privacy and data sovereignty ( Hummel *et al.*, 2018), which goes against the grain of the current model of data extraction and storage by corporations. It was a process of sociotechnical construction whose core was not primarily the design or even the test of a technology (which certainly played a role), but rather, the co-creation of a narrative involving a community.

The Open University of Catalonia, as a part of the DECODE consortium, ran the DDDC pilot between 2018 and 2019. The participatory, online-offline process was oriented to collectively imagine an alternative data economy and society while implementing DECODE technology. The pilot took place for 6 months and was developed in several phases, running between October 2018 and April 2019. Throughout the process, different face-to-face meetings were held, connected with the activity developed on the Decidim platform.

The three key expected outputs of the pilot can be reframed in terms of the technological, narrative, and social dimensions mentioned earlier: first, a technological output was developed to test the integration between the DECODE architecture and the Decidim platform ( Sagarra *et al.*, 2019); secondly, a textual output created to test the collaborative development of a critical narrative around data; and lastly, a sociotechnical result to generate a public debate and a community around these issues.

## Co-creation approach for drafting a manifesto on data commons: community, technologies and narrative

For the specific methodology for the creation of the manifesto, drawing on the need to combine different knowledge backgrounds and perspectives from scholars and practitioners in the context of the DDDC pilot, we describe and analyse below the co-creation process of a manifesto as a sociotechnical process. The main strategy was to establish a collaborative discussion and writing process that could make previous conversations around the DECODE project crystallize in a shared narrative, which afterwards could be published online within the Decidim platform, therefore entering a second stage of discussion, support and dissemination. The methodological rationale of this approach was twofold: first, applying participatory design as a key practice of co-creation ( Sanders & Stappers, 2008) to manage the diversity and complexity of transdisciplinary collaboration ( Sanders *et al.*, 2010), and specifically adapt it to the experimental format of “writing sprints” ( Hawkins, 2016) and “writing retreats” ( Benvenuti, 2017). Second, opting for a manifesto as an interaction-based outcome, considering it from a co-creation perspective as a performative textual and narrative format ( Ashby *et al.*, 2017; Hanna, 2019). Such an approach can bridge diversity of knowledge as a social impact and communication strategy ( Freedman, 2018). Our methodologies for this manifesto co-creation served to explore and build a shared vision (rather than merely putting a pre-existing one into words), and also contributed to going beyond the paradigm of the “expert-author”, by opening the process up to a variety of actors. This final stage of open sharing brought together a network of collectives interested in the issues addressed by the manifesto, in a way that not only the object (the manifesto itself) but also the subject (the related network), co-emerged during the mentioned DDDC pilot process.

The collaborative writing of the DDDC Manifesto had three main phases. First, an open debate with the participation of communities of experts and stakeholders. Second, a co-writing sprint manuscript, led by DECODE partners (from Internet Interdisciplinary Institute at Universitat Oberta de Catalunya), based on the outcomes of the debate, and finally, a manifesto open public signature and dissemination phase. The outcomes and results of the process are presented and described in this section following the three core aspects of the DDDC pilot, under a threefold sociotechnical perspective: (1) “Community”: participants, knowledge base and overall engagement process, (2) “Technologies”: manifesto co-creation process, techniques and tools used, and (3) “Narrative”: content analysis and use of the resulting manifesto. 

Derived from the overall community engagement process and the content generated in the two preliminary workshops described above, the DDDC Manifesto co-writing sprint represented a challenge to structure the necessary narrative, following a coherent participatory approach. Dotson (2017) highlights that community exists along a spectrum, from 'thin' forms of association to 'thick' ones, where individuals strongly identify with the group. In the case of data commons, understanding this range is key to fostering participation. The community we engaged with reflected a range of interactions, from 'thin' membership, characterized by casual participation, to 'thick' membership, where individuals viewed their involvement as crucial to their identity and the commons' success. Understanding this spectrum was vital to navigating the challenges of creating a cohesive manifesto. The starting point, in terms of content generated until that stage, was a core structure according to three axes previously defined (legal, economic and governance aspects around data commons) and required to consider a total of 82 concrete proposals gathered via Decidim for each of the three mentioned axes. Departing from the premise of generating a new text from scratch following a manifesto format, it was necessary to establish key interaction bases that could allow for both the integration of the outcomes of the DDDC pilot and its elaboration, in an iterative and distributed way. For this reason, the manifesto sprint opted for a co-creation dynamic in which various rounds of brainstorming, writing, discussion and review of the text could take place while being developed. Specifically, in order to connect the needed narrative to a process of collective intelligence and creative “commoning”, we chose to articulate convergence and divergence sequences as a basic principle grounded in design thinking ( Sanders & Stappers, 2008), unfolded iteratively for the development of the first manifesto draft.

### Basic principles for a collaborative writing sprint

Adopting an approach that dates back to the principles of Open Space Technology ( Owen, 2008), the sprint started with the setting of a flexible workspace where participants could interact together, consisting of a central area with empty wall space for sticky notes and visualizations, and a large main screen to visualize progress. Another prerequisite was to have comfortable “writing corners” with tables and chairs to work autonomously, either individually or as separated clusters, when needed, to write in smaller groups. Another key element in setting the approach and philosophy of the sessions for the two-days sprint, was to display a series of core principles on a small poster with the “Norms of the space for a manifesto sprint”, which stated: “(1) Writing is (even) more important than talking!; (2) Reading (our own work) is more important than writing; (3) We are aiming at a manifesto (not an article, nor a book); (4) Let's respect timing and iteration phases”. Besides these principles, the establishing of a main facilitation role was also required, adopted by one of the participants to guide the whole process, confirm time frames for each stage, clarify or unblock when necessary during the interactions, and to moderate discussions in the various “convergence” phases. Considering both the setting of the space and the physical artefacts (walls, posters, sticky notes, etc.) as key “soft-technology” resources, this preliminary preparation allowed for the unfolding of a co-creation process guided by principles of shared capacity, reflexivity and flexibility. 

### “Warm-up” brainstorming about data futures

**Figure 2.**
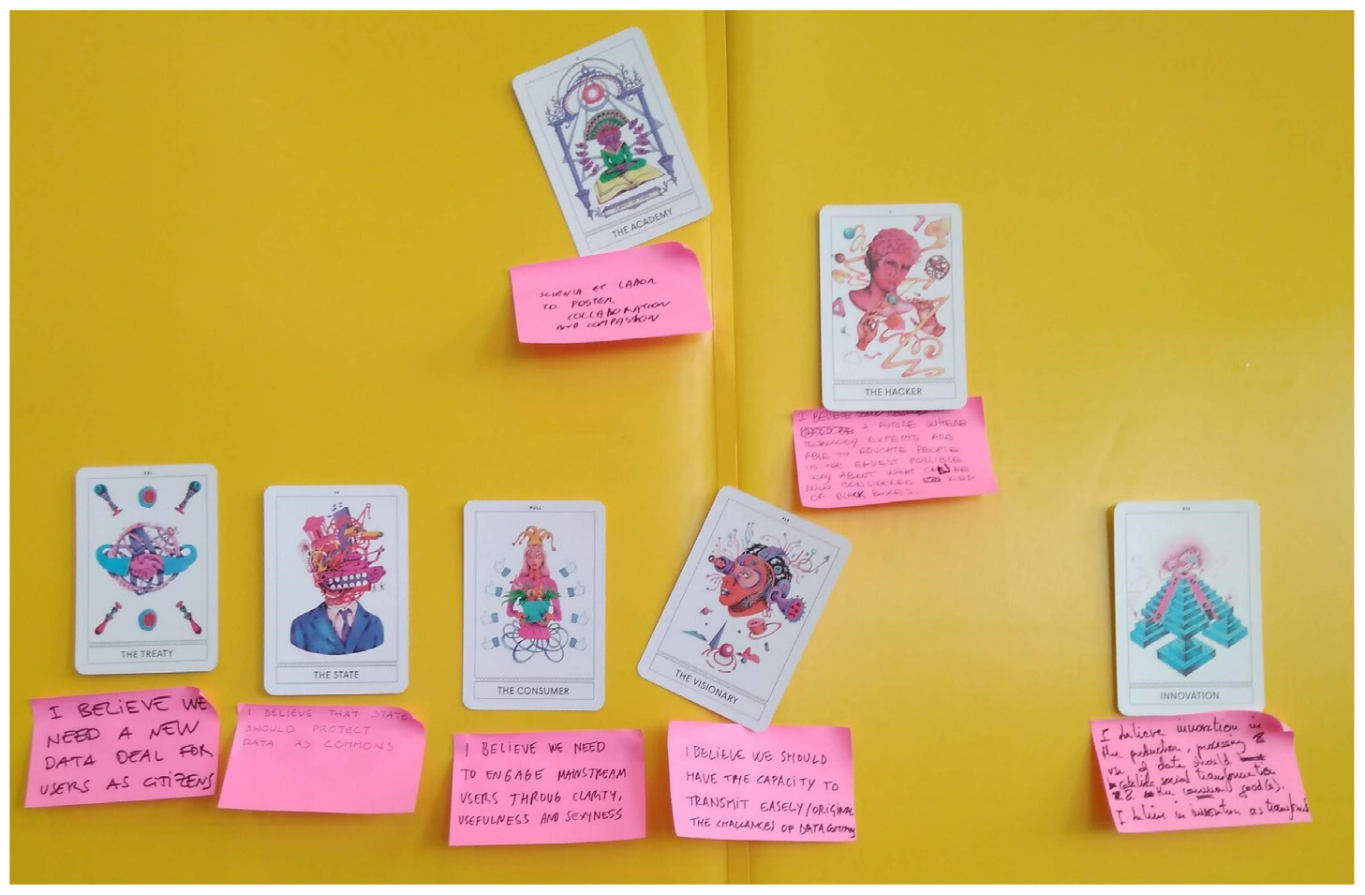
Results of the interaction using Instant Archetypes.

Before addressing the collaborative writing process itself, it was also necessary to start the sprint stating the importance of taking into account both personal viewpoints and shared visions regarding the topic, as well as a “breaking the ice” technique for discussing and addressing challenges around data commons. For this, a short interaction was conducted among participants at the very beginning of the sprint, consisting in adapting the use of the “Instant Archetypes” toolkit, containing different cards with roles and positions regarding digital and data-related futures. Using this interpretation of the major Arcanes of the tarot deck, as used in previous studies ( Desjardins *et al.*, 2020), participants could express personal beliefs in short sentences about prospective scenarios under several diverse viewpoints, such as “The State”, “The consumer”, “The Academy” or “The hacker”, among others ( Figure 2).

Once various ideas about possible futures were made explicit by each participant by choosing one of the 20 available cards to visualize a specific viewpoint, a discussion was organized to try to homogenize the different personal visions. Reconstructing the phrase “I believe...” used for each card with a shared narrative under the formula “We believe...”, this was articulated in coherence with the need to depart from plural imaginaries and perceptions about digital technologies, whilst at the same time, including other key issues beyond data (such as power, knowledge, freedom, labour, etc).

### Anatomy of a manifesto

Afterwards, as another necessary preliminary step to set the co-writing process, diverse aspects of the structure and content of various manifestos on similar topics were discussed. This was necessary not only for participants to be more familiar with such textual and discourse format, but also to keep adding new perspectives on technology and data-related narratives. Starting from a list previously prepared by some of the participants, among several positioning texts around the question of data (with similar or discordant positions), the main focus was on the “ Manifesto in favor of technological sovereignty and digital rights for cities”, the “ MyData Declaration” and “ The Data-Centric Manifesto”.

This approach also allowed for participants to familiarize themselves with the core language and characteristics of these manifestos, from their basic structure (in terms of introduction, diagnosis, goals and specific calls to actions) to their descriptive or normative approach, as well as references to actors or narrative styles (such as more aphoristic or argumentative ones, for example).

### State of the art in previous DDDC proposals

**Figure 3.**
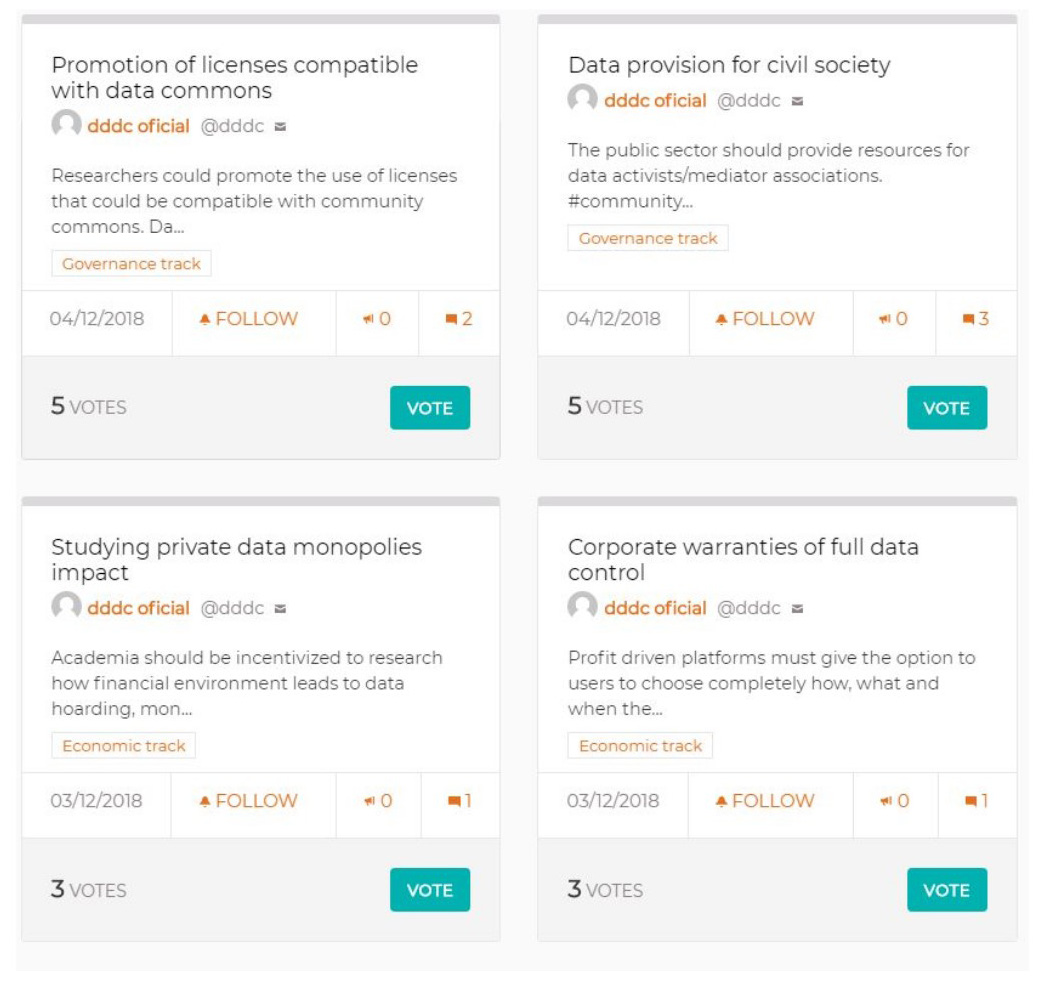
Sample of four cards with previous proposals for the manifesto (source: Decidim platform).

Another necessary step, to connect with the previous DDDC community engagement and co-creation processes, consisted of a phase in which all contributions in the form of DDDC proposals generated until then could be taken into account for the manifesto. For this, based on the ideas from participants in the two previous workshops, as well as the period of open online participation through the Decidim tool mentioned above, a total of 82 paper cards were printed with their title and description ( Figure 3). This allowed their ordering and the prioritizing of those which could provide important ideas and insights during the elaboration of the manifesto, by targeting key issues as well as specifying key policies or principles to take into account for the final structure of the text.

These cards, extracted and printed from the Decidim online interface, represented an opportunity to convert concepts from a digital format into an offline one, where they could be rearranged and re-appropriated in order to gain wider significance and coherence under a co-creation approach.

### Generative writing phase: sequence of divergence

The co-writing process began with a division into small work groups based around a different set of cards and their DDDC proposals. In this way, there could be an initial autonomous development of content according to the identified areas in relation to data commons: legal, economic and governance aspects. For this, groups of 3 or 4 participants were allocated in different areas of the space and each one discussed and sought to develop previous contents, using a synthetic format of “bullet points” and lists of concepts. The groups first worked on their own digital document on a digital PAD, as a co-creative phase of divergence, which allowed adding as many ideas as possible. Subsequently, prior to merging the different sections into a single digital document (in order to move forward in an integrated way), each group “rotated” to another table to review and try to improve or expand what had been written. This type of informal “peer-review” was based on the “World Cafe” methodology ( Steier *et al.*, 2015), where a member of the original group remained in place to present and clarify what was previously written and decided in that specific section of the manifesto draft.

### Cohesive writing phase: sequence of convergence

After repeating another writing review sequence, at the end of the first day there were different texts as subsections developed on each pad. These were merged on a single digital document, which served as a starting point for the next sprint day. This second, cohesive writing phase started with a visualization of the manifesto draft based on the open source Voyant Tools text analysis software, which allowed participants to have an overview of keywords, recurrences, and general structure of the manifesto as generated the previous day. Afterwards, a complete version of the manifesto draft was printed on large format paper, with identical copies placed on different tables, allowing different groups of participants to continue working in parallel on the text via analogical reading and paper annotations. 

At the end of the second sprint day, after digitalizing the annotations and conclusions derived from the parallel review of the manifesto draft, a final group discussion took place with two experts in data commons and digital policies as external participants. This allowed the group to focus on the introductory section of the manifesto and to identify necessary improvements prior to publication. Then, a final version of the text was agreed, needing subsequent “touch-ups” online (for a limited time in the following weeks), but already with a broad consent for its publication and dissemination.

## Results of process and the DDDC Manifesto

The DDDC pilot described consisted of a six-month process, and a variety of actors were involved as a result. The community of participants began to coalesce at a preliminary “kick off workshop” that took place in October 2018 at Fabra & Coats (Barcelona), in the context of the event “Digital Cities, Digital Freedoms: Digital Commons, Ethical Standards and Free Software for Cities”. The gathering was a starting point for a participatory diagnosis about the state of legal, governance and economic models currently emerging around data, supported by analyses previously developed in the context of the DECODE project. This initial phase allowed us to address the novelty and complexity of knowledge areas intersecting with the topic of data commons. This first workshop engaged 35 participants to discuss and confirm the need to advance, in parallel, in the three main areas: legal, economic and governance perspectives. Based on the diagnosis of the initial meeting, a second preliminary workshop was organized during the Sharing Cities Summit, in November 2018, which generated additional proposals for the three mentioned axes of the DDDC pilot (legal, governance and economic). Proposals which emerged during that co-creation process were added to the Decidim digital platform to be debated and voted on. Working in specific areas, 223 participants and platform users were able to include 82 additional proposals for these axes as well as general ones, which as described constituted the raw content for the subsequent manifesto sprint. The final online document consisted of a 1,363-words corpus of text with three main sections, for which we reflect here some of the most relevant excerpts:

### Diagnosis

- “Today, everything can be turned into data. Digital technology is deeply changing the world and the way we work, learn, move, share, decide; even the way we love”. [...]
- “Free software, hardware, knowledge, and culture point towards a digital society without artificial scarcity, grounded on a logic of fairness and cooperation rather than exploitation and competition”. [...]
- “There is a need to advance from open data to data commons, from “my data” to “our data”. Data commons mean data of, by and for the people”.

### Beliefs, principles and values

- “Privacy and data protection are key: furthermore, people have the right to be free from all forms of unlawful or unfair interference in their digital life”. [...]
- “Data should primarily nurture emancipatory social transformation instead of unjust perpetuation, blind disruption or mere innovation”. [...]
- “Corporations should respect people’s digital life and rights worldwide, the State should work for this, and the people ascertain it”. [...]

### Goals and actions

- (Rights and society. Constructing a well-regulated and fair digital society) “Develop and promote technological, legal and practical tools that provide privacy, security, and ethics by design”. [...]
- (Democracy. Building an augmented democracy) “Generate spaces where citizens can participate in data-based research agendas, specially, based on public data”. [...]
- (Economy. Moving towards a commons-based digital economy) “Incentivize the development and scope of digital platforms that do not base their sustainability in user’s data exploitation, but in a commons oriented sustainability model”. [...]

To observe and reflect the evolution of the manifesto’s narrative between the different phases of co-creation, we performed a contrast of content versions. To do so, we used again Voyant Tools, which allowed us to focus on how different key aspects of the content evolved after the initial and final drafting of the manifesto, rather than focusing on issues such as number of sprint participants, their origin or transdisciplinary background.

**Figure 4.**
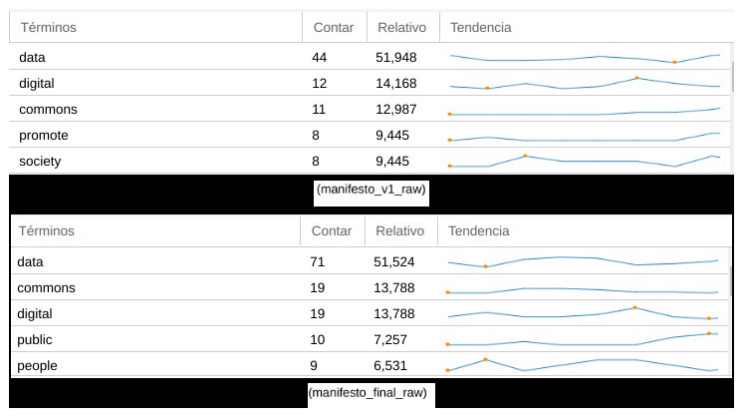
Comparison of recurrent terms in two versions of the manifesto.

When we compare the final version of the manifesto (as agreed and published after the co-writing sprint) with previous versions of the draft (in the preliminary stages of collaborative writing on PADs), some relevant factors could be observed around the evolution of its dimension, structure and narrative. A first consideration when we analysed textual content and discourse between previous and final versions was how key concepts stood out from the rest ( Figure 4), reinforcing their presence in the case of the words “data” (from 44 concurrences in the draft to 71 in the final version), “digital” (from 12 to 19) and “commons” (from 11 to 19). Subsequently, the other most relevant terms used in the final version of the manifesto were “public” (10 concurrences) and “people” (9 concurrences), above terms used more often in preliminary versions such as “promote” (8 concurrences) and “society” (8 concurrences), in a sort transition from an administrative style to one of political agency –of claiming public responsibility beyond abstract demands. This also suggests that the manifesto’s final content elaboration contained more clear and accessible language than in its preliminary draft, reflecting how the co-creation process in relation to narratives and discourse moved progressively away from a scholarly and policy-oriented tone.

**Figure 5.**
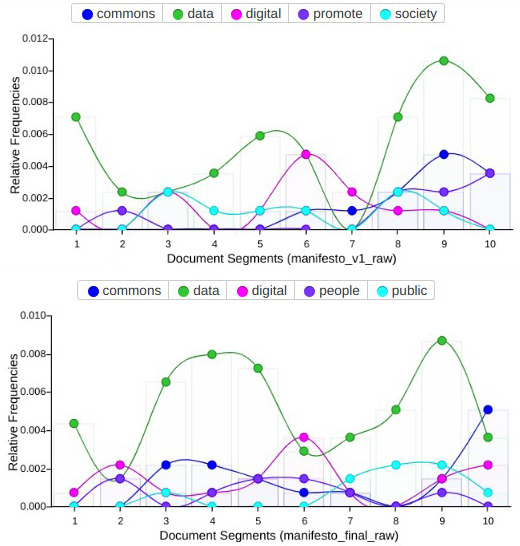
Evolution of the most frequent terms in both versions of the manifesto.

A general visualization of the frequency in which these terms were distributed throughout both versions of the manifesto in ( Figure 5), also suggested that the term “data” was used more prominently and recurrently in the final one. This changed from a few lines to 847 words in the draft during the sprint manifesto, and afterwards 1,378 words in its final version. Another order of comparisons related to how the initial tripartite topic structure evolved. Focused on the issues of legal and rights, socio-economic context, and governance and democratizing aspects, with just a brief introduction, the draft version moved to a much more cohesive document structure in the final version of the manifesto, containing short sentences rather than initial convoluted ones, as well as a more direct tone.

Regarding key new concepts in the final version, the positions and proposals around data commons were preceded by a more detailed diagnosis after the first sprint interactions. For example, when proposing the need to advance in the key area of the network society, clear allusions were made to challenges regarding social control, economic benefit of data, technological determinism and sustainable socio-economic alternatives, among others. An initial section on “Beliefs, principles and values” also stood out in the final structure of the manifesto, preceding the concrete proposals derived from the participatory process of the DDDC pilot. Specifically, these were positions regarding the need to advance in the context of data commons, guided by principles of freedom, equality and sorority, with a desire for processes of augmented democracy, and a sustainable data-based economy respectful to people’s rights.

The manifesto’s final version, after the co-writing sprint ( https://datacommons.barcelona/our-vision/), was presented to the DDDC Final meeting in April 2019. This public event included the open final stage of its digital signing, as the first testing of an alpha version of the DECODE-Decidim technology. It was followed by an open debate with scholars and experts in the field of technology policies and digital economy.

## Discussion

This section considers some of the key debates posed by the DDDC pilot process and the resulting DDDC manifesto, highlighting its possibilities and limits. A first key topic relates to the variety of ways for approaching the development of sociotechnical systems. Crucially, the DDDC pilot was a process of sociotechnical co-creation whose core was not primarily the design or even the test of a technology (which certainly played a role), but rather, the co-creation of a narrative and a community. Furthermore, the co-created narrative was about a technological society, rather than a specific or set of technologies. This implied going beyond techno-centric approaches in sociotechnical construction and placing, at its centre, the relationship between narratives and actors. Debates around frames and scripts, not on concrete technologies but on technological paradigms, seem crucial at this moment, in which conceptual and theoretical frameworks around data are still in their infancy. Furthermore, to activate alternative imaginaries represents a crucial approach in the current predicament, where multiple crises seem to haunt existing models of society, politics and economy. To an extent, the DDDC pilot process was a way to counter Jameson’s famous quote where “it is easier to imagine an end to the world than an end to capitalism”. As Morozov (2015) and Fisher (2009) also argue, imagining non-capitalist futures in a heavily commercialized data economy is a significant challenge. The manifesto seeks to explore such alternatives, though this remains a work in progress. The point was to imagine another possible world in relation to data, a non-capitalist one, where projecting and debating alternative technological societies is as crucial today as building alternative technologies, since they are part of the same process of transformation. 

However, it is probably not enough to push only for alternative visions. As our case study reflects, the DDDC approach should not only be understood as a challenge to technocentric approaches to digital developments, but also to more traditional, narrative and socio-centric forms of social transformation. DDDC and the resulting data commons manifesto put technologies, and not just its social aspects, as a key element for debate. Furthermore, it seeks to provide a political vision of technologies and the social, where the manifesto collaborative writing was itself part of the development of a technology. The key point here can be formulated by rephrasing the traditional Kantian formula around the relations between concepts and intuitions: technologies without narratives are blind, while narratives without technologies are empty.

There is a third key element in the construction of sociotechnical systems relating to this case: the social part, driven by community building, which shaped, and was shaped by, both the technology and the narrative. In this sense, the creation of both technologies and narratives emerged from communities around DECODE and Decidim. This brings us to an additional key point for discussion, the specific form of constructing sociotechnical systems deployed in DDDC: the use of methodologies of co-creation and participation, which put the social and the community at its centre. Community engagement strategies are crucial for raising awareness and addressing the shortcomings of both the existing digital economy and the possibilities of data commons. For this, the principles and methods adopted from participatory design that we described, when applied to the “collaborative writing sprint” format, effectively contributed to outline an alternative narrative of sociotechnical systems, connecting a variety of perspectives that went beyond these in order to succeed. In this sense, having anchoring points, in this case with legal, governance and economic dimensions, was key for advancing understanding and awareness in a wider data commons narrative. Beyond this, the manifesto format afforded a type of participation more open than a purely “policy-oriented” process, in that a wider diversity of people could connect to the political debates that emerged in parallel. As noted before, a final key result of this type of participatory process, guided by co-creation methods, was the constitution of the local Barcelona Data Commons network, an informal link between various Barcelona-based organizations working in the field of data commons, from theoretical to applied perspectives, and from the university to the private sector.

A further key aspect, related to the issue of participation, was the “emergentist” approach adopted for the DDDC pilot. The three elements in sociotechnical construction (technologies, narratives and communities), and in particular those of narrative and community, were not ready-made but a result of the co-creation process itself. The narrative did not begin with a set of co-authors with a vision, or an existing community that wanted to reach further, as has usually been the case in modern or “traditional” manifestos. Instead, at the beginning of the DDDC process the vision and the community already existed, but were not explicitly formulated. Only a set of preliminary elements and actors were there in advance, coming from the DECODE project, which meant a broad starting point (unknown for most participants), where the DECODE project members (as part of a pre established consortium) took the role of facilitators rather than “experts”. As a result, a sort of collective authorship emerged in the process of building a narrative, a vision, and the technology itself that could implement part of it. The relation between the subject and the object were tinkered through co-creation within the same process, as we have tried to describe.

However, while proving their potential to construct and perform narratives, technologies and communities, the approach in the DDDC pilot also showed several limits, either of principle or practice. Narratives, technologies and communities are key elements in social transformation, but they are difficult both to create, align and sustain. Explicitly multiplying the number of factors under construction for articulating a shared discourse, as exemplified in the manifesto sprint, increases the resources required and widens the probabilities of narrative consistency. In the case of DDDC, the success on each of these fronts could be considered asymmetric. A technology was tested, but only in an alpha version that would require extra resources to be fully functional. Beyond that, there is also the core issue of the wider adoption of the DDDC pilot. On the narrative side, the manifesto took a powerful rhetorical form, but its internal structure still displays flaws: the listed policy proposal currently has a weak connection with earlier parts, for example. Also, some proposals lack a principle of organization and the action points remain unclear, so overall, the final version of the text does not follow a fully consistent development. Much of this has to do with the participatory nature of the process of its drafting, as well as the time limits framed by the context of a project with different aspects, participants and especially deadlines, which can set clear areas of discourse but can also affect others in its intention or extension.

There were also limits of participation itself. A key one had to do with selection bias, which can take a quantitative form (total number of people in relation to the ideal number of people) and a qualitative form (social profiles excluded from, or not sufficiently enticed by the process). This selection is especially significant regarding technology, when mostly “white, male, middle-age, middle-high income” profiles tend to predominate. Regarding the selection bias in the case of the DDDC pilot, more than 200 people participated in the overall process, and the data we have suggests that while there was no gender disparity, other gaps, such as racial disparities, were patently present. A second limitation is participation bias, that is, the balance between different participants, internal power dynamics and various ways of engaging in practice. Even though the DDDC spaces were mediated and oriented forms of equal participation, certain actors, especially those of the DECODE consortium, played a central role throughout the process, not only as facilitators, but also as content producers. In that sense, the break with the “expert” figure was less clear than desired. Finally, a third participation hurdle related to complexity. Participation implies bringing in a variety of voices with different perspectives and levels of knowledge, and for the successful development of a multi-authored text in this case, during the face to face manifesto sprint, limitations in number and type of participants collecting and writing according to the 82 received proposals were evident as well (12 sprint participants in total, through the overall process previous to online sharing). On the other hand, even with a proper structure, it is difficult to manage the social and, in this case, complexity of data as a topic. As a result, the manifesto can be seen as still unconcluded and far from a perfect piece both in rhetorical and policy terms, and therefore as a sort of “beta version”.

## Conclusions

The present paper presents and analyzes the results of a collaborative writing sprint carried out in the framework of the DDDC pilot (DECODE project). The methodological approach to the study stems from the theory of manifestos and deliberative and participatory democracy, with special emphasis in the contributions of the theory of sociotechnical systems, commons and co-creation. Though the DECODE-Decidim project concluded in 2019, its principles of decentralized governance and data sovereignty remain highly relevant in the face of contemporary challenges posed by AI and algorithmic systems. As AI technologies increasingly disrupt various sectors and fuel concerns around data exploitation and surveillance, the principles embedded in the Digital Democracy and Data Commons Manifesto offer a technopolitical counter-narrative that emphasizes human-centric and democratic data governance.

The Digital Democracy and Data Commons Manifesto transcends its role as a mere textual document, functioning as a technopolitical artefact designed to challenge the dominant AI-driven and data-extractive models of governance. By advocating for decentralized and commons-based alternatives, the manifesto offers a blueprint for community-oriented data systems that resist the technocentric and extractivist logics of current platforms. The writing sprint represented an effort to gather the needs expressed by participants, at different stages, to increase privacy and data sovereignty, facing the current model of data extraction and exploitation by big tech corporations. The phases for its creation were consistent with divergence and convergence iterations typical in participatory design, moving from an informal corpus of content to structured sections and specific ideas and claims in the resulting text.

As a result, the co-creation of the manifesto was not only able to integrate the previous outcomes of a participatory process via the Decidim platform, and via specific workshops with diverse participants, but also to evolve through several versions in a relatively short time, which ended up in a structured corpus of an alternative narrative addressing shared visions about data commons.

Among the main limitations of our case study, beyond the complexity of adopting comparative approaches with other similar cases (due to the novel methodological and technical process adopted and described here), the main one was selection bias regarding the viability of including all previous participants into the two-days sprint, due to its face to face and intensive nature. While the manifesto provides a robust rhetorical foundation, we recognize that a more detailed alignment between its narrative and actionable steps would improve its uptake. The challenge lies not just in the content but in transforming these ideas into lived sociotechnical realities that engage communities at a deeper level. We acknowledge that constructing alternative sociotechnical imaginaries requires more than articulating ideas on paper. The success of such efforts lies in fostering continuous engagement and community practices that embody these ideals, linking policy to everyday data governance practices. Indeed, Purtova & van Maanen (2023) emphasize the importance of aligning data governance models with practical outcomes that address the specific challenges of data capitalism. The manifesto attempts to bridge this gap, though further work is required to align its vision with policy. Indeed, recent works on digital sovereignty and urban data governance have highlighted the importance of commons-based frameworks in addressing the challenges posed by smart city infrastructures and AI-driven data economies. For instance, Calzada (2021) emphasizes the significance of ‘data sovereignty’ in smart cities as a means to resist data extractivism and promote participatory urban governance. Scholars like Fuchs (2021), Morozov (2015), and Ostrom (1990) also underline the importance of participatory models that challenge the extractivist practices of big tech and offer viable alternatives for community-centered data governance.

In this sense, however, we consider the manifesto and the results of the process that led to its collaborative writing, as a sort of sociotechnical narrative commons in practice, and therefore as an open outcome -both regarding this article and the manifesto itself.

## Data Availability

No data are associated with this article.
