# Supplementary material for: Co-creation of the Digital Democracy and Data Commons Manifesto: alternative sociotechnical visions of data
Source: Open Res Eur. 2024 Oct 25;4:45. Originally published 2024 Mar 1. [Version 2] doi: 10.12688/openreseurope.17020.2 (PMC11589416; doi:10.12688/openreseurope.17020.2)
Supplement: Peer review history

# Reviewer report: Reviewer response for version 2

**DOI:** 10.21956/openreseurope.20313.r45620

**Published:** 2024-12-14

**Authors:** Carla Bonina

**Affiliations:** 1 University of Surrey, Guildford, England, UK

This article delves into the collaborative creation of the Digital Democracy and Data Commons (DDDC) Manifesto under the EU Horizon 2020 DECODE project. It highlights the importance of participatory design and data governance that prioritizes community control, ethical considerations, and democratic participation. The authors detail the co-creation process involving in-person and digital practices, leading to a manifesto advocating for transitioning from data extractivism to a commons-based model. By framing the manifesto as a technopolitical artifact, the paper contributes to ongoing debates about digital sovereignty and sociotechnical imaginaries.

 I thoroughly enjoyed reading the article. My review acknowledges that the Open Letter has undergone several rounds of peer review, which provided valuable feedback for improvement. The authors have already incorporated critical suggestions, such as enhancing theoretical and case contextualization, integrating key literature, and clarifying the Manifesto’s relevance. I found the section on “Co-creating Visions” particularly robust and very informative.

 The following minor suggestions aim to further enhance the manuscript.

 Diversity and Inclusivity in Participation. One key theme that I commend in the article has to do with the integration of community, technological, and narrative dimensions in the co-creation process, and its focus on inclusivity and participation. The paper documents that this process involved challenges (which are common in participatory designs); the manuscript rightly acknowledges the different selection, participation and complexity bias in the discussion, highlighting a gender balance but racial and socioeconomic gaps among participants. I was particularly curious to understand the participant demographics, and how the participants were invited/selected at different stages in the project. Adding this point could add value to other participatory process, and I’d suggest the authors to explain briefly how the invitation and selection was done, as well as a summary of the profile of the participants over time. A clear fit would be in the Co-creation approach for drafting a manifesto…” section.

 In addition, because the selection bias is highlighted as a main limitation, it’d be interesting to outline those specific lessons learned in the recruiting or selection process that could be helpful to mitigate the bias in future processes.

 On the Sociotechnical Design and the Co-Existence of Face-to-Face and Online Co-Creation Dynamics. The manuscript does a very good job of describing how the Decidim platform was used in conjunction with other participatory practices conducted outside digital means. The conclusions could include a brief note about future work, suggesting the provision of more detailed examples of specific functionalities of Decidim that supported participatory design and how these functionalities reinforced the manifesto's feasibility and relevance in data governance debates. By doing so, it could contribute more broadly to the extensive literature on online deliberation and illustrate how the process of co-creation may shape its outcomes.

 Overall, I find the piece engaging and with the potential to spark debate in a growing and highly relevant area of data governance. Its integration of participatory methods with sociotechnical systems design is insightful, and the focus on building an inclusive process is commendable. The suggestions offered above are intended to refine specific aspects of the manuscript and enhance its impact on both academic and practical discussions.

Where applicable, are recommendations and next steps explained clearly for others to follow? (Please consider whether others in the research community would be able to implement guidelines or recommendations and/or constructively engage in the debate)

Partly

Does the article adequately reference differing views and opinions?

Yes

Are all factual statements correct, and are statements and arguments made adequately supported by citations?

Yes

Is the rationale for the Open Letter provided in sufficient detail? (Please consider whether existing challenges in the field are outlined clearly and whether the purpose of the letter is explained)

Yes

Is the Open Letter written in accessible language? (Please consider whether all subject-specific terms, concepts and abbreviations are explained)

Yes

Reviewer Expertise:

Data governance, digital platforms, digital inclusion, policy

I confirm that I have read this submission and believe that I have an appropriate level of expertise to confirm that it is of an acceptable scientific standard.

# Reviewer report: Reviewer response for version 2

**DOI:** 10.21956/openreseurope.20313.r46870

**Published:** 2024-12-06

**Authors:** Claude Draude

**Affiliations:** 1 University of Kassel, Kassel, Germany

The Open Letter reconstructs and discusses the six-month development process that resulted in the "Digital Democracy and Data Commons Manifesto". The manifesto development was conducted as part of the EU-funded DECODE (Decentralized Citizen Owned Data Ecosystems) project and gives insight into the Barcelona Data Commons network. 

 The authors' intention with the Open Letter is two-fold: tracing the writing sprint that enabled contributors from various backgrounds to co-create the manifesto as well as analyzing how the co-creative and iterative process shaped and altered the content structure of the manifesto.

 The Open Letter seeks to illustrate how collaborative, participatory design approaches can address pressing concerns related to datafication and empower citizens in the digital sphere through data commons. The evolvement from manifesto to technopolitical artefact which is retraced in the revised Open Letter is particularly interesting.

 The Open Letter has already been subject to several rounds of peer-review that provided valuable feedback and comments for improvement. In the current version, suggestions such as integrating key literature, providing contextualization etc., have been incorporated and led to an improved version of the original paper. 

 At this stage, I have only a few minor suggestions that could be integrated to enhance readability and understanding, particularly for a broader audience.

1. The introduction starts with the problem scope and then switches to the manifesto and DECODE project.  The project is then explained in the DDDC pilot section. For people unfamiliar with the background it would be beneficial to introduce the DECODE project and background when first mentioned, at least briefly.
2. Links to the Communist Manifesto and Accelerationist Manifesto would increase accessibility of the Open Letter.
3. The link to the DDDC Manifesto appears to be broken.
4. Further references have been included as a response to previous reviews. The use of references and the explanations of concepts is executed at different levels of depths, however. In line with the Open Letter format my suggestion is to go through the manuscript once more and make sure that concepts are not only substantiated through literature but also briefly explained. Examples are "design thinking" or, more importantly, because it is central to your approach, "participatory design". 
5. Questions of power and the redistribution of power, in particular, are central to participatory design practices. While you mention power dynamics in your paper this aspect could be taken up in the conclusion and made more prominent. 
6. The discussion and conclusion section could connect back to, or further develop, some of the arguments and findings introduced in the introduction. This would enhance the discussion and conclusion by providing a more critical and in-depth analysis—not only of the collaborative writing sprint but also of this (or similar) approaches in the broader context of a datafied society.

Where applicable, are recommendations and next steps explained clearly for others to follow? (Please consider whether others in the research community would be able to implement guidelines or recommendations and/or constructively engage in the debate)

Partly

Does the article adequately reference differing views and opinions?

Yes

Are all factual statements correct, and are statements and arguments made adequately supported by citations?

Yes

Is the rationale for the Open Letter provided in sufficient detail? (Please consider whether existing challenges in the field are outlined clearly and whether the purpose of the letter is explained)

Yes

Is the Open Letter written in accessible language? (Please consider whether all subject-specific terms, concepts and abbreviations are explained)

Partly

Reviewer Expertise:

Sociotechnical systems design; participatory design; design applications, methodologies and theories for more inclusive, socially just digital systems

I confirm that I have read this submission and believe that I have an appropriate level of expertise to confirm that it is of an acceptable scientific standard.

# Reviewer report: Reviewer response for version 2

**DOI:** 10.21956/openreseurope.20313.r45628

**Published:** 2024-11-25

**Authors:** Matthew Hanchard

**Affiliations:** 1 iHuman Institute (Faculty of Social Sciences), The University of Sheffield, Sheffield, England, UK

The paper is well-written and has clearly been through several rounds of proofing and peer-review. It presents Decidim, DECODE, the DDDC manifesto (and the need for it) alongside the emergence of the Barcelona Data Commons network very well.

 However, I might have expected a little more on the wider context in which a manifesto might sit. For example, there is no mention of the OSTP’s Nelson Memo, or COAlition S’s Plan S, UNESCO 'Recommendation on Open Science’ , or even the recent Barcelona declaration. These all direct contemporary research policy towards open access data (forming a particular policy landscape), and could easily have been drawn on by the author(s) to evidence the significance the paper itself more forcefully – and with it, the need for a manifesto and more emergentist and participatory ways of working.

 Similarly, a broader discussion on the role of open data amidst a shift in the way science and democracy are configured, could have perhaps drawn on discussion of longer-standing movements towards open source, open access, open research, and open science in general – including the politics of the copyleft movement vs. a standardisation of data repositories. At times these are hinted at, with head-nods – but then never quite followed up. Their importance is all-the-more-so when the author(s) speak of extractivism and the notion of a commons (with the spectre of capitalism hinted at, but never fully drawn out). Other manifestos discussed in the paper do this specifically and explicitly as political intervention. Instead, democracy (deliberative or otherwise) is spoken of in methodological terms, and linked to participation at the level of epistemics and not ontology. This leaves the paper feeling a little descriptive, and lacking depth in terms of the author(s) voice and personal politics; it is a thesis *for* participation in which the author(s) hold back on their own participation.

 A short paragraph in the discussion section could have summarised the above and linked them back to the introductory section, adding voice and personality. Within the author(s)’ own framework – which I might add, is very well articulated in the Introduction section – this indeed could have leant on Jasanoff (and Kim) to work around sociotechnical imaginaries, or looked to place a data commons manifesto as a niche intervention within a particular sociotechnical regime or configuration of open data, or even offered a Latourian mapping. In any new/revised version drafted, that would certainly make a welcome addition – but it is a ’nice to have’, not a necessary or required change for me.

 Otherwise, there are no major faults in this paper. It sets out its aim, charts a theoretical approach and methodology, discusses the research, and presents an argument for a manifesto and a commons. In doing so it meets its promises well, and with an appropriate level of rigour. While it is tempting to nit-pick faults just to ‘prove my worth’ as a reviewer, doing so would be entirely unnecessary – the paper is sound as it is, and has clearly already been strengthened by addressing the previous round of reviewers’ comments. There is little more I can add, and I shall look forward to reading future work by the author(s)

***References***

 Barcelona Declaration on Open Research Information (2024) Barcelona Declaration on Open Research Information. doi: 10.5281/zenodo.10958522. 2024 (Ref - 1)

 cOAlition S (2024) *Addendum to the coalition s guidance on the implementation of plan s: Principles and implementation*, *Plan S*. Available at: https://www.coalition-s.org/addendum-to-the-coalition-s-guidance-on-the-implementation-of-plan-s/principles-and-implementation/. 2024 (Ref -2)

 European Commission (2024b) *The EU’s open science policy*, *Strategy 2020-2024: Our digital future*. Available at: https://research-and-innovation.ec.europa.eu/strategy/strategy-2020-2024/our-digital-future/open-science_en. (Ref - 3)

 Jasanoff, S. (2015 [Ref - 4]) ‘Future imperfect: Science Technology, and the Imaginations of Modernity’, in Jasanoff, S. and Kim, S.-H. (ed.) *Dreamscapes of modernity: Sociotechnical imaginaries and the fabrication of power*. London: University of Chicago Press.

 OSTP (2022 [Ref - 5]) *Memorandum for the Heads of Executive Departments and Agencies: Ensuring Free, Immediate, and Equitable Access to Federally Funded Research*. Washington, DC.

 UNESCO (2021 [Ref - 6]) ‘Recommendation on Open Science’. Paris. doi: 10.54677/MNMH8546.

Where applicable, are recommendations and next steps explained clearly for others to follow? (Please consider whether others in the research community would be able to implement guidelines or recommendations and/or constructively engage in the debate)

Yes

Does the article adequately reference differing views and opinions?

Yes

Are all factual statements correct, and are statements and arguments made adequately supported by citations?

Yes

Is the rationale for the Open Letter provided in sufficient detail? (Please consider whether existing challenges in the field are outlined clearly and whether the purpose of the letter is explained)

Yes

Is the Open Letter written in accessible language? (Please consider whether all subject-specific terms, concepts and abbreviations are explained)

Yes

Reviewer Expertise:

Open access, Digital society, Digital sociology, Research Methodology (including Participatory methods), and Science and Technology Studies (STS).

I confirm that I have read this submission and believe that I have an appropriate level of expertise to confirm that it is of an acceptable scientific standard.

## References

[1] Barcelona Declaration on Open Research Information (2024) Barcelona Declaration on Open Research Information.2024; 10.5281/zenodo.10958522 10.5281/zenodo.10958522 Reference source

[2] Addendum to the coalition s guidance on the implementation of plan s: Principles and implementation, Plan S.2024; Reference source

[3] European Commission (2024b) The EU’s open science policy, Strategy 2020-2024: Our digital future. Reference source

[4] : Future Imperfect: Science, Technology, and the Imaginations of Modernity. *Chicago: University of Chicago Press* .2015; 10.7208/9780226276663-001 1-33 10.7208/9780226276663-001 Reference source

[5] : Memorandum for the Heads of Executive Departments and Agencies, August 25, 2022: Ensuring Free, Immediate, and Equitable Access to Federally Funded Research.2022; Reference source

[6] : UNESCO Recommendation on Open Science.2021; 10.54677/MNMH8546 10.54677/MNMH8546

# Reviewer report: Reviewer response for version 2

**DOI:** 10.21956/openreseurope.20313.r45515

**Published:** 2024-11-21

**Authors:** Gijs van Maanen, Charlotte Ducuing, Tommaso Fia

**Affiliations:** 1 Tilburg University, Tilburg, North Brabant, The Netherlands; 2 KU Leuven, Leuven, Flanders, Belgium; 3 University of Tübingen, Tübingen, Germany

Thank you for the revised version in which you accommodated our feedback. We think that the authors revised their paper in a satisfactory manner, and we especially appreciate the changes made in the conclusions of the paper.

 We have several comments that have to do with the location in the text of newly added sentences. We are not sure whether the section starting with “While the process aimed for inclusive participation …” is integrated well. Please make sure that the sentences before and after this insertion are well-connected to the included sentences. Please do the same thing for the added section on Dotson (starting with “Dotson (2017) highlights that…”). Here it feels like these sentences are directly inserted in the former text without changing the flow of the text and the order of the themes discussed there. The authors indicate that ‘understanding this spectrum was vital to navigating the challenges of creating a cohesive manifesto’. Yet, the paper does not clearly state how this would be indeed the case concretely. Alternatively, the reference to the thin / thick community spectrum may also consist of an analysis lens leveraged only a posteriori, which is not necessarily a bad thing either but would also have to be expanded. In short, the reference to the community spectrum, as it is, looks a bit artificial and contrived. Perhaps further reflections on Dotson’s distinction could find more space in the conclusions section, to better integrate it within the overall analysis. Lastly, the sentence “Indeed, Purtova & van Maanen (2023) emphasize the importance of aligning data governance models with practical outcomes that address the specific challenges of data capitalism. The manifesto attempts to bridge this gap, though further work is required to align its vision with policy.” (from the Conclusions section) could be fleshed out to include a warrant against top-down, paternalistic approaches to policymaking which impose ideal models of data commons based on different contexts and experiences. This adjustment would be in line with the paper’s commitment to democratic participation and deliberation.

Where applicable, are recommendations and next steps explained clearly for others to follow? (Please consider whether others in the research community would be able to implement guidelines or recommendations and/or constructively engage in the debate)

Yes

Does the article adequately reference differing views and opinions?

Yes

Are all factual statements correct, and are statements and arguments made adequately supported by citations?

Yes

Is the rationale for the Open Letter provided in sufficient detail? (Please consider whether existing challenges in the field are outlined clearly and whether the purpose of the letter is explained)

Yes

Is the Open Letter written in accessible language? (Please consider whether all subject-specific terms, concepts and abbreviations are explained)

Yes

Reviewer Expertise:

Political theory; Science and Technology Studies; data governance

We confirm that we have read this submission and believe that we have an appropriate level of expertise to confirm that it is of an acceptable scientific standard.

# Reviewer report: Reviewer response for version 1

**DOI:** 10.21956/openreseurope.18393.r39846

**Published:** 2024-08-14

**Authors:** Igor Calzada

**Affiliations:** 1 Cardiff University, Cardiff, Wales, UK

This paper tackles the timely and crucial topic of data commons, offering insights into its potential significance amidst contemporary sociotechnical landscapes. While the paper introduces an important contextual topic, namely data commons, the integration of references appears somewhat lacking in thoroughness. Nevertheless, given the potential contribution to the ongoing literature review on data commons, the article merits consideration for indexing.

 A notable aspect that requires attention is the positioning of data commons within the broader literature. The manuscript could benefit from a more comprehensive integration of existing literature on the subject to enhance its contextualization. Furthermore, the reference to DECODE-Decidim, though relevant, prompts questions regarding the currency of the research, considering the project's historical nature. Clarification from the author regarding how their manifesto addresses contemporary concerns surrounding AI disruption and paranoia would be valuable.

 The suggestion to present data commons not only as a manifesto but also as a technopolitical artefact offers an intriguing avenue for exploration. Such an approach could provide an alternative perspective to the prevailing technocentric, AI-driven global narrative. Accordingly, I recommend that the manuscript undergo a thorough revision to address these points and strengthen its positioning within the current discourse.

 Additionally, the absence of key literature on the topic is a notable gap that warrants attention. Including relevant literature would enrich the scholarly contribution of the paper and provide readers with a more comprehensive understanding of the subject matter.

 The abstract effectively highlights the paper's focus on addressing concerns regarding the proprietary and exploitative use of personal data, framing digital commons as a potential alternative. The experimental approach outlined, aimed at co-creating the Digital Democracy and Data Commons Manifesto, demonstrates a commitment to principles of openness, diversity, and inclusivity.

 However, the abstract could benefit from clearer delineation of the specific outcomes of the collaborative writing sprint and the subsequent analysis of the manifesto's evolution. Providing more detailed insights into the methodology and results would enhance the abstract's comprehensiveness and clarity.

 In conclusion, while the paper shows promise in addressing pertinent issues surrounding data commons, several areas require refinement to strengthen its scholarly contribution and relevance. With revisions to improve the integration of references, address historical context, and enhance clarity and comprehensiveness, the manuscript has the potential to make a valuable addition to the literature on this important topic.

Where applicable, are recommendations and next steps explained clearly for others to follow? (Please consider whether others in the research community would be able to implement guidelines or recommendations and/or constructively engage in the debate)

Partly

Does the article adequately reference differing views and opinions?

Partly

Are all factual statements correct, and are statements and arguments made adequately supported by citations?

Partly

Is the rationale for the Open Letter provided in sufficient detail? (Please consider whether existing challenges in the field are outlined clearly and whether the purpose of the letter is explained)

Partly

Is the Open Letter written in accessible language? (Please consider whether all subject-specific terms, concepts and abbreviations are explained)

Partly

Reviewer Expertise:

technopolitics, datafication, social innovation

I confirm that I have read this submission and believe that I have an appropriate level of expertise to confirm that it is of an acceptable scientific standard, however I have significant reservations, as outlined above.

## References

[1] : Data commons. *Internet Policy Review* .2024;13(2) : 10.14763/2024.2.1748 10.14763/2024.2.1748

[2] : Barcelona's grassroots-led urban experimentation: Deciphering the 'data commons' policy scheme. *Zenodo* .2019; 10.5281/zenodo.2604618 10.5281/zenodo.2604618

# Response

**Published:** 2024-10-17

**Authors:** Ricard ESPELT

Thank you very much for your insightful and constructive feedback on our manuscript, "Co-creation of the Digital Democracy and Data Commons Manifesto: Alternative Sociotechnical Visions of Data." We appreciate your detailed comments and suggestions, which have helped us to improve the manuscript significantly. Below, we address each of your points in detail, outlining the changes we have made to strengthen the integration of key literature, clarify the historical context, and enhance the overall clarity and contribution of the paper. 1. Integration of References and Contextualization of Data Commons You noted that the manuscript could benefit from a more comprehensive integration of existing literature on data commons to enhance its contextualization within broader academic discourse. We fully agree with this suggestion and have taken steps to address this. Changes made:

- We have expanded the literature review in the Introduction and Discussion sections to integrate key works on data commons, digital sovereignty, and governance. These include foundational works by Elinor Ostrom (1990), as well as more recent studies by Fuchs (2021), Morozov (2015), and Purtova & van Maanen (2023). These scholars provide a broader socio-political framework that emphasizes the need for commons-based approaches to data governance.
- Additionally, we have incorporated Igor Calzada’s (2021) work on smart cities and data sovereignty, which aligns well with the paper’s focus on community-driven governance. Calzada’s emphasis on urban data commons as an alternative to extractive models in smart city environments strengthens the manuscript’s connection to contemporary issues in data governance.

Relevant Text Added: "In recent years, scholars have increasingly focused on data commons as a critical framework for addressing inequalities and governance challenges in digital ecosystems (Ostrom, 1990; Fuchs, 2021; Morozov, 2015). Furthermore, Calzada (2021) emphasizes the need for ‘data sovereignty’ in urban settings, presenting the concept of urban data commons as a viable alternative to the extractivist practices of AI-driven smart cities. These contributions provide a robust theoretical foundation for our exploration of data commons as a technopolitical response to contemporary governance challenges." Reference added:

- Calzada, I. (2021). Data co-operatives through data sovereignty. Smart Cities, 4(3), 1158-1172..

2. Clarification of the DECODE-Decidim project's relevance You raised a valid point regarding the historical nature of the DECODE-Decidim project and its relevance to contemporary concerns, particularly surrounding AI disruption and paranoia. We have revised the manuscript to clarify how the manifesto's principles remain pertinent to current challenges. Changes made:

- We added a paragraph in the Discussion section that addresses how the outcomes of the DECODE-Decidim project continue to be relevant today, particularly in light of emerging concerns about AI, surveillance, and algorithmic governance.
- The revision links the manifesto’s advocacy for data commons and decentralized governance to current debates about AI’s impact on privacy and the growing need for data sovereignty.

Relevant text added: "While the DECODE-Decidim project was completed in 2019, its principles of decentralized data governance and data sovereignty remain highly relevant in addressing contemporary concerns surrounding AI disruption and data paranoia. As AI technologies increasingly automate decision-making processes and exacerbate data-driven surveillance, the manifesto’s call for community-centered and commons-based governance presents a technopolitical response to these pressing challenges." 3. Reframing the Manifesto as a technopolitical artefact The suggestion to present the data commons manifesto not only as a manifesto but also as a technopolitical artefact offered a valuable opportunity to broaden the scope of the paper. Changes made:

- We have reframed the manifesto in the Conclusion as a technopolitical artefact, emphasizing its potential role as a living document that challenges the dominant technocentric, AI-driven governance models.
- This revision underscores the manifesto’s capacity to inspire and guide policy development, digital infrastructure, and participatory technology design.

Relevant text added: "The Digital Democracy and Data Commons Manifesto transcends its role as a mere textual document and functions as a technopolitical artefact that challenges the dominant AI-driven and data-extractive models of governance. By advocating for decentralized and commons-based alternatives, the manifesto offers a practical blueprint for constructing community-centered data systems that resist the technocentric logics of current platforms." 4. Enhancing the abstract for clarity and specific outcomes You noted that while the abstract effectively highlights the focus of the paper, it could benefit from clearer delineation of the specific outcomes of the collaborative writing sprint and subsequent analysis. Changes made:

- We revised the abstract to include more specific details about the outcomes of the collaborative writing sprint, the methodology used, and the evolution of the manifesto.

Revised abstract: "This paper presents the co-creation of the Digital Democracy and Data Commons Manifesto through a collaborative writing sprint, drawing on principles of openness, diversity, and inclusivity. The manifesto articulates a technopolitical vision for data governance that prioritizes community control over data. We analyze the manifesto's evolution throughout the process, demonstrating its capacity to address contemporary concerns such as data extractivism and algorithmic governance." 5. Incorporation of key literature Finally, you highlighted the absence of key literature on the topic. We have taken steps to address this by incorporating several influential studies that reinforce the paper’s theoretical foundation. Changes made:

- We added key references to works by Ostrom (1990), Fuchs (2021), Morozov (2015), Purtova & van Maanen (2023), and Igor Calzada (2021). These references have been integrated throughout the manuscript, particularly in the Introduction and Discussion sections, to strengthen the contextualization of data commons and their relevance to current digital governance challenges.

Relevant text added: "Scholars have increasingly focused on data commons as a framework for addressing inequalities and governance issues in digital ecosystems (Ostrom, 1990; Fuchs, 2021; Morozov, 2015; Purtova & van Maanen, 2023). Calzada (2021), in particular, highlights the significance of urban data commons as a tool for promoting data sovereignty in smart cities, presenting an alternative to extractive models driven by AI and big data."

# Reviewer report: Reviewer response for version 1

**DOI:** 10.21956/openreseurope.18393.r39839

**Published:** 2024-06-03

**Authors:** Gijs van Maanen, Tommaso Fia, Charlotte Ducuing

**Affiliations:** 1 Tilburg University, Tilburg, North Brabant, The Netherlands; 2 University of Tübingen, Tübingen, Germany; 3 KU Leuven, Leuven, Flanders, Belgium

**Introduction:**

 In times when data are considered one of the key ingredients of oppressive and exploitative regimes of, among others, ‘data capitalism’ and ‘platform capitalism’, ideas, initiatives and models on how to govern data better have gained prominence in both academic, public, and commercial spheres of society. 

 The article written by Hidalgo *et al. *can be considered as part of these discussions. What makes Hidalgo *et al.*’s contribution interesting and relevant in light of the growing interest in data governance, is its explicit focus on and reflection about the kind of ‘theory of change’ needed when attempting to put data governance in practice, namely, one brought about by changes in how we think and talk *about *technology. The authors, in other words, argue for a reversal, or at least a problematization, of the latent techno-determinism still often present in data sharing, governance, or donation initiatives. Hidalgo *et al.*'s starting point is that not only technology, but also rules, infrastructures, stories, and mutual expectations are key to take into consideration and study when researching our sociotechnical reality (Hidalgo et al, p. 1).

 By thus going beyond *both *the mere formulation of models, frameworks, and theories, *and* tech and data-deterministic forms of thinking, Hidalgo *et al.* offer a valuable contribution to the EU data governance debates in relation to local (digital) democracy (Fischli & Muldoon, 2024; Micheli et al., 2020).

 In the remainder of this review, we take you step by step through Hidalgo *et al.*’s argument and highlight several pointers for further discussion, debate, and research.

**Sociotechnical imaginaries:**

 One of the methodological starting points of the paper is the idea that you cannot understand (and in a second step, change) technological systems without paying attention to ‘social’ actors and dimensions. ‘Sociotechnical systems’ is the term used in the field of ‘science and technology studies’ (STS) to draw attention to the more-than-technological dimensions of technology. Crucial here, for Hidalgo *et al.*, are the widely shared beliefs and visions about technologies and data that support tech companies in their abilities to easily gather, store, and trade data. You need stories about the relevance and value of data, the argument seems to be, to be able to make a living from it. This interrelationship and interdependence of the social in the form of narratives and imaginaries, and data and technology, is for Hidalgo *et al.* sufficient reason to argue that “(...)  that transforming sociotechnical systems requires the articulation of alternative narratives and imaginaries.” (p. 3) Just like you need stories to convince people of the relevance of data, you need alternative stories to make them change their mind. 

 While the need to invoke stories and imaginaries when attempting to instantiate societal change is an important point, it is not clear entirely what this would amount to within the specific context of Hidalgo *et al.*’s article. Who was the audience of the project and manifesto (more on this below) and what did they think about the influence of data and technology?

 What kinds of beliefs about the importance of for instance data commons did they already have (or not)? Were those involved tech-enthusiasts without too many worries about the influence of commercial actors on our societies, or did they already agree with the author’s problem analysis that there are genuine problems with respect to the governance of data, and these can best be approached via democratic and commons-based approaches?

 These are important questions to answer because they will specify the problem to be dealt with - is it really one of a lack of consensus about the problematic nature of big tech, or something else? - and subsequently, the kind of solution best tied to that specific problem-analysis - is that one of narrative-change or the transformation of such narratives into policy? Answers to such questions will be informative for the specific function attached to the co-creation of the manifesto, and hence the success of the project.

**Digital democracy, data commons, and community:**

 Hidalgo *et al.’*s project was part of a 6-month pilot called Digital Democracy and Data Commons (DDDC), that was part of the larger EU funded DECODE (‘Decentralized Citizen Owned Data Ecosystems’) project organized in Barcelona. DDDC aimed to nurture online and offline discussions by citizens on digital infrastructures, and the collective authoring of a data commons manifesto. Hidalgo *et al.*’s paper discusses this last exercise: the writing of the manifesto.

 The authors situate the project within a longer tradition of Manifesto-writing - from the *Communist Manifesto* in the 19th century, to Haraway’s *Cyborg Manifesto* in the 1980s - and ask how such texts could function as “cultural and symbolic resources that can create new avenues for collective action,” and are also capable of creating “a sense of community based on mutuality and rationality.” (p. 4) The writing and publishing of manifestos could thus be of relevance, the authors suggest, for projects like digital and data commons that aim to develop more sustainable and democratic forms of data production, use, and governance.

 Hidalgo *et al.* discuss in detail how they organized the collective writing process of their data commons manifesto. Over a period of six months, in several writing and discussion ‘sprints’, and by making use of various thinking tools and techniques, all participants (a total number of 200 individuals who participated in the process) managed to compose a manifesto which can be found here: ( https://datacommons.barcelona/our-vision/; last accessed May 2 2024).

 This was, unexpectedly, a complicated and time-consuming process. Many different individuals during different phases needed to be able to have some sort of input into the collaborative writing exercise. That such a process is also fraught with difficulties relating to who and what kind of participants were in the end capable of influencing the process is for readers with some familiarity with deliberative democratic procedures unsurprising. As the authors themselves note in their discussion, limitations with respect to the desired degrees of representation in the group of participants were noticeable. Experts members of the DECODE consortium, for instance, had more of a say in the content that was being produced during those moments. What could have been clarified in the author’s analysis of the process is the extent to which experts have shaped co-creation of the manifesto itself. From the brief overview of the various writing phases, an open debate involving ‘communities of experts and stakeholders’ preceded the co-writing sprint that formed the core of the article’s analysis (p. 6). However, the results of such an open debate are not overtly discussed. Even more fundamentally, it is not evident who qualified as a ‘stakeholder’, nor is discussed the extent to which the outcome of the open debate has shaped and influenced as a blueprint the subsequent phase of co-writing sprint. Moreover, is not explained how the commons ended up as a solution to the problems under consideration. All these questions are worth paying attention to when trying to grasp which actors influenced the writing process in what way.

 Possibly more interesting from the perspective of theories on the digital and data commons, is however the author’s usage of the label of ‘community’ to designate the group of people involved in the collaborative process. As is being argued in the conclusion of the paper, 

 “Crucially, the DDDC pilot was a process of sociotechnical co-creation whose core was not primarily the design or even the test of a technology (which certainly played a role), but rather, *the co-creation of a narrative and a community*.” (p. 11) [emphasis added]

 The usage of the concept of community within discussions on commons specifically, and tech development in general, is prevalent. As such, the associations made by the article between ‘commons’, ‘groups’, ‘collectives’, and ‘community’ is understandable and from a practical point of view probably also desirable. Words and narratives matter for how technology is supposed to work, as the authors explained, and how an initiative is described (or framed) matters for its success.

 How the relationship between commons and community is to be understood is, however, a complicated question discussed in both empirical and philosophical literature. In an exchange going back almost 30 years, commons scholars Elinor Ostrom and colleagues Sara Singleton and Michael Taylor discussed the relevance of attaching the label of ‘community’ to a commons (Ostrom, 1992; Singleton & Taylor, 1992). To what extent does ‘community’ add to our understanding of what a commons could mean? Ten years later, political theorist Elizabeth Frazer placed her doubts on the often vague and ambiguous usage of the word community by her ‘communitarian’ peers (Frazer, 1999). Why use the word ‘community’ for describing a ‘neighborhood’ when it does not add much to what the latter concept already conveys? What do, we henceforth ask, Hidalgo *et al.* try to convey with their usage of ‘community’ when describing their project?

 In general, we think that there is room for ‘community’ in the ‘commons’, although the usage of this term does deserve scrutiny and specification. Network scholar Taylor Dotson identifies seven dimensions of ‘community’, located on a spectrum ranging from very ‘thin’ forms of ‘community’, to very ‘thick’ ones (Dotson, 2017). Communities whose members, for instance, have a very strong self-identification with that community can be located on the ‘thick’ end side of the spectrum. For such individuals their membership is of existential importance and leaving the community (‘exit’) is almost unthinkable for them (cf. Marglin, 2010). For members of more ‘thin’ communities, by contrast, membership of their community is comparatively speaking less important, often characterized by more contract-based and infrequent interactions, and leaving the community is accordingly also relatively easy and burdenless. Community, in other words, has different dimensions, related to different types of living or working together.

 The work of Dotson invites to be specific about the kind of community one is talking about within the context of digital or data commons, in order to better understand the kind of commons one is talking about. How much of the identity of members of the common depends on the existence of the said common? How important is its flourishing for them? Questions like these shed light on what one can expect of the members of a commons, but also on the narratives and imaginaries needed to be able to create these senses of collective self-identification.

 And this allows us to move our discussion from the processes to create a manifesto, to the manifesto’s content. How is the manifesto tied to what kind of understanding of community, and subsequently, to what kind of idea on what the data commons ought to be?

**The data commons manifesto:**

 The manifesto starts by concisely listing the often-heard harms and dangers present in our digitized economies: the datafication of everything, the lack of control we have about our data and about who controls ‘our data’, the inequalities, precarisation, et cetera., all caused by the unequal redistribution of the value extracted from data, resulting in the degradation of democracy and the environment.

 The manifesto calls for the development of ‘strong data commons’: data commons that are not merely or only premised on openness (of data, and access to data), but that also take data production, protection, governance, and the ‘social responsibility’ for their impact into account. Data commons are more than individual data control, and have ‘collective dimension’.

 In several subsequent sections, the manifesto unpacks the direction data commons should have to go. Key values like freedom, equality, fraternity and sorority should guide the development of  a “radical and augmented democracy”, where “everyone is allowed, able and willing to rule, and where collective intelligence, deliberation and action are boosted.” Commercial actors should ‘respect’ this, states check whether they do so, and citizens should keep an eye on their political representatives.

 More concretely, the manifesto makes cases for several related policy proposals, among others:

 The co-construction of a ‘model of data commons’ where is ensured that the interests of all involved parties are reconciled in a “trustworthy, privacy-protection and mutually value-producing way.”

- The regulation, monitoring, and auditing of data by the public sector, with a specific emphasis on interoperability, data portability, data sovereignty.
- Tools to provide privacy, security, and ethics by design, as well as better privacy agreements.
- The promotion of data ecosystems.
- More democratic governance models and data-based citizen participation.
- Educational efforts to increase citizen’s ‘data-awareness’.
- The empowerment of people through the advancement of data control, data sharing, and data exploitation.
- The promotion of free and creative common licenses that also contribute to “personal and community rights,” various data-related citizen science projects.
- And projects revolving around a more ‘commons-based’ digital economy, including, sustainable digital platforms, open data, ‘commons-oriented business’, and ‘commons communities and ecosystems.’

And not to be forgotten here, is the manifesto’s call to go ‘beyond data’: “(...) data is only a small piece in such endeavors. People and life are their centers.”

 For readers familiar with the different ways in which - both historically and more recently - the digital and data commons have been understood, a variety of strands in the commons literature can be read into the manifesto (Purtova & van Maanen, 2023). One can recognise the arguments from the commons-based peer production literature about the importance of knowledge sharing for democracy. One can see the more radical interpretations of the commons that argue for strict separations between market, state, and society/commons. Also noticeable are the strands in the literature that do think there is value in citizens themselves to extract the value of data as a means to ‘take back some control’ over those digital means of production. And woven through those different calls to arms, are proposals to strengthen democratic institutions with the help of digital tools, citizen participation, and citizen-based knowledge production.

 The manifesto, in other words, captures nicely and concisely the great plurality of ways in which the digital and data commons can be understood. Because of this, the manifesto’s scope has the potential to attract a wide audience. Precisely for this reason, readers of Hidalgo *et al.*’s paper could be a bit at loss when trying to grasp the manifesto’s uptake. While the authors write in the conclusion that the ‘core issue’ was “the wider adoption of the DDC pilot,” they seem to explain this by referring to the ‘internal structure’ of the manifesto: 

 “(...) the manifesto took a powerful rhetorical form, but its internal structure still displays flaws: the listed policy proposal currently has a weak connection with earlier parts, for example. Also, some proposals lack a principle of organization and the action points remain unclear, so overall, the final version of the text does not follow a fully consistent development.” (p. 12)

 The question to be asked about this analysis is whether the cause of the apparent lack of uptake of the manifesto can fully be explained by the content of the manifesto. Or, in other words, how much is to be expected from sentences on a piece of paper? And how does one translate those sentences into indeed the stories and imaginaries that are needed - according to the author’s theory of change - to bring about the desired sociotechnical change in the world. A more encompassing reflection on the extent to which the product of the project - the manifesto - can be considered an alternative ‘sociotechnical imaginary’, would have been appreciated. Note that this question does not warrant a binary answer; the question asks in the end how one construes a narrative that is not put on paper, but also lived by people in practice.

 Relatedly, but from a different angle, is the question of whether the authors of the manifesto have rightly identified the issue at stake that should be solved with the help of a new 'imaginary’. For instance, the power of big tech companies derives from, among many other things, complicated legal constructs like Intellectual Property that are quite resistant to beliefs and stories. How citizenry stories should relate to these more rigid dimensions of our digitized universe (if at all) is worth unpacking.

 Lastly to be noted, is the broader question of why data commons should be the solution to many if not all of the problems listed. *If* one, for instance, thinks that the problem is best characterized by ‘data capitalist’ practices of profit-oriented data-gathering, does one wants to keep that extractivist model and simply be in control of it through better data governance, or is one interested in getting rid of that issue altogether by imagining a non-capitalist future (cf. Morozov, 2015)? The different strands of commons literature noticeable in the manifesto – from reformist ones, to more revolutionary ones – are illustrative of the immense difficulty of imagining a world so radically different from our current one (cf. Prainsack, 2023; Fisher, 2009).

**On manifestos and narratives:**

 This brings us to the question - via a long detour - with which this section started: what kind of community was present in or through the manifesto, and how did this invoke a type of data commons?

 As suggested above, the manifesto catered for a broad variety of interpretations of what the digital and data commons could mean. Many different groups, collectives, and communities would be able to see something reminiscent of their beliefs and theories about how a better digital society should look like in the manifesto. That is, arguably, also its strength.

 At the same time does the text lack - these are obviously our speculative reflections - a *concrete audience*, a *community*. The problems and solutions presented are *everyone’s*, and therefore, to a certain extent, *no one’s* (cf. Fisher, 2009, p. 66). What might be missing here is what Ostrom once called the ‘CPR situation’ (Ostrom, 1990, p. 191). Ostrom talked about CPR situations within the context of a discussion on the relationship between theoretical models, and actually existing forms of collective action in the form of commons. As she writes, “Clear analytical models provide an important part of the theoretical foundation for good policy analysis, but not the entire foundation.” (Ostrom, 1990, p. 191). And while 

 “[m]odels suggest to the analyst likely behaviors and outcomes in a situation with a *particular *structure. They do not tell the analyst how to discover the structure of the situation in order to conduct an analysis.” (Ostrom, 1990, p. 191)

 The usage of relatively high-level models and theories - the ones many of us start our papers with (‘data capitalism’, ‘informational capitalism’, et cetera) - lack, by virtue of their theoretical nature, a more concrete analysis of what this then means for those affected by it, which are within the context of commons then also those burdened with the task to do something about the problem. Similarly, are solutions presented to these problems - data governance theories and models - equally abstract. What, in other words, does the manifesto and the subsequent establishment of Barcelona Data Commons network, mean for those who participated in the process? What are the problems to be dealt with there, and in what way do these present a CPR situation motivating common governance?

 These reflections warrant asking the question of how to translate the often abstract arguments (models?) for data commons into stories that allow people to recognize the ‘CPR situation’ they’re in, and the subsequent need to engage in practices of commoning. The question is thus not whether Hidalgo *et al.* are right in thinking that narratives are crucial for sociotechnical change - we think they raise an important dimension of good tech governance here. The question is rather what kind of narratives, told by whom, to whom, when, and where, allow citizens to recognize their specific CPR situation, and convince and motivate them to act accordingly. A good narrative, it almost goes without saying, is only part of the story. Sustainable data common governance is a multi-dimensional endeavor requiring - next to a good story about its raison d’être - the right legal, political, communal, etc. conditions to become a success. Important here to consider are the situation-specific relationships between those different elements: a good narrative without the proper infrastructure for data storage and usage won’t result in much of a success; the presence of a high tech data sharing technologies without a convincing story for why it will be of use will not do much good either. A proper analysis of what precisely the good is that is to be governed in common is crucial; this, notwithstanding, to avoid developing solutions for a type of problem better to be solved in a different, possibly less-communal way (Ducuing *et al.*, 2024; Purtova & van Maanen, 2023). 

**References:**

 Dotson, T. (2017). *Technically Together: Reconstructing Community in a Networked World*. The MIT Press.

 Ducuing, C., Fia, T., & Maanen. (2024). Legislating on Data Commons: What It Should (Not) Be. *Platform Cooperativism Consortium*. https://platform.coop/blog/legislating-on-data-commons-what-it-should-not-be/

 Fisher, M. (2009). *Capitalist Realism: Is There No Alternative?* Zero Books.

 Fischli, R., & Muldoon, J. (2024). Empowering Digital Democracy. *Perspectives on Politics*, 1–17. https://doi.org/10.1017/S1537592724000409

 Frazer, E. (1999). *The problems of communitarian politics: Unity and conflict*. Oxford University Press.

 Marglin, S. A. (2010). *The Dismal Science*. Harvard University Press.

 Micheli, M., Ponti, M., Craglia, M., & Berti Suman, A. (2020). Emerging models of data governance in the age of datafication. *Big Data & Society*, *7*(2), 2053951720948087. https://doi.org/10.1177/2053951720948087

 Morozov, E. (2015). Socialize the Data Centres! New Left Review, 91, 45–66.

 Ostrom, E. (1990). *Governing the Commons: The Evolutions of Institutions for Collective Action*. Cambridge University Press.

 Ostrom, E. (1992). Community and the Endogenous Solution of Commons Problems. *Journal of Theoretical Politics*, *4*(3), 343–351. https://doi.org/10.1177/0951692892004003006

 Prainsack, B. (2023). The Roots of Neglect: Towards a Sociology of Non-Imagination. TECNOSCIENZA: Italian Journal of Science & Technology Studies, 13(2), Article 2.

 Purtova, N., & van Maanen, G. (2023). Data as an economic good, data as a commons, and data governance. *Law, Innovation and Technology*, *0*(0), 1–42. https://doi.org/10.1080/17579961.2023.2265270

 Singleton, S., & Taylor, M. (1992). Common Property, Collective Action and Community. *Journal of Theoretical Politics*, *4*(3), 309–324. https://doi.org/10.1177/0951692892004003004

Where applicable, are recommendations and next steps explained clearly for others to follow? (Please consider whether others in the research community would be able to implement guidelines or recommendations and/or constructively engage in the debate)

Yes

Does the article adequately reference differing views and opinions?

Yes

Are all factual statements correct, and are statements and arguments made adequately supported by citations?

Yes

Is the rationale for the Open Letter provided in sufficient detail? (Please consider whether existing challenges in the field are outlined clearly and whether the purpose of the letter is explained)

Yes

Is the Open Letter written in accessible language? (Please consider whether all subject-specific terms, concepts and abbreviations are explained)

Yes

Reviewer Expertise:

Political theory; Science and Technology Studies; data governance

We confirm that we have read this submission and believe that we have an appropriate level of expertise to confirm that it is of an acceptable scientific standard, however we have significant reservations, as outlined above.

# Response

**Published:** 2024-10-17

**Authors:** Ricard ESPELT

We sincerely appreciate your valuable feedback on our manuscript. After reviewing your comments and suggestions, we have made significant revisions to the paper, addressing your concerns in the following areas. Please find below a detailed response to your points, organized by section, along with a description of the changes we have implemented.

 1. Clarification of the theory of change (Introduction) You pointed out that while the paper introduces an important idea of a "theory of change" for data governance, it was not clear how this concept operates within the specific context of the article.

 Changes made: We have expanded the introduction to clarify the proposed "theory of change," linking it to the need for alternative sociotechnical imaginaries. Specifically, we emphasize that the paper challenges the dominant techno-deterministic approaches and proposes a participatory model that integrates narratives, infrastructures, and shared expectations, as Micheli et al. (2020) and Fischli & Muldoon (2024) argue, data governance requires governance frameworks that promote collective control and democratic participation. This revision ensures that readers understand how the model can be applied in practice.

 2. Role of participants and community engagement (sociotechnical imaginaries) You asked for more detail on the role of participants in the manifesto process, questioning what pre-existing beliefs they had about data commons and how these were accounted for during the project.

 Changes made: We have added a paragraph in the Sociotechnical Imaginaries section that explains the diversity of the participants involved in the manifesto process. Specifically, we now discuss how the participants ranged from tech-enthusiasts to data commons advocates, each bringing a different perspective on data governance. This diversity shaped the narrative development, ensuring that the process challenged both complacency and corporate influence on data governance. We have also drawn on Dotson’s (2017) spectrum of ‘community’ to highlight the varying levels of commitment participants had to the commons-based approach.

 3. Expert influence on the process (Digital Democracy and Data Commons) You pointed out that the influence of experts, particularly from the DECODE consortium, could have been more thoroughly analyzed, especially concerning how they shaped the manifesto's outcome.

 Changes made: We added a more in-depth reflection on the role of experts in the process, acknowledging that while the process aimed for inclusive participation, expert stakeholders from the DECODE consortium played a central role in shaping the technical frameworks. This influence was particularly evident during initial debates, which established the manifesto’s structure. However, the revision clarifies that efforts were made to ensure equal contribution from all participants, including non-experts, throughout the co-writing process.

 4. Concept of community in Data Commons (Community and Commons concept) You requested clarification on how the concept of community was used in relation to commons and suggested drawing on philosophical and empirical literature.

 Changes made: We have elaborated on the use of "community" in the Community and Commons Concept section, referencing Taylor Dotson’s (2017) seven dimensions of community. We now specify that the engaged community ranged from "thin" forms of casual involvement to "thick" forms where individuals viewed their participation as vital to the commons’ success. This allows a clearer understanding of how the community was constructed during the process and how it influenced the manifesto.

 5. Manifesto’s structure and practical impact (Manifesto Content and Narrative) You highlighted the need for reflection on whether the manifesto’s structure contributed to the uptake or if more practical elements were required to translate the narrative into practice.

 Changes made: In response, we expanded the Manifesto Content and Narrative section to reflect on the connection between the manifesto’s structure and its practical impact. We acknowledge that while the manifesto provided a strong rhetorical foundation, its internal structure could benefit from clearer links between narrative sections and policy proposals. Drawing on Purtova & van Maanen (2023), we emphasize that further work is needed to align the manifesto’s vision with actionable governance models to ensure its successful adoption. 6. Integration of bibliographic references We have incorporated the bibliographic references suggested by the reviewers to strengthen the theoretical foundation of the paper. Here is where we integrated each reference:

- Micheli et al. (2020) and Fischli & Muldoon (2024): These references were added in the introduction to support the argument that data governance requires more than just technical solutions. They help frame the article’s focus on governance frameworks that promote collective control and democratic participation.
- Ostrom (1990) and Prainsack (2023): These references were included in the Sociotechnical Imaginaries section to support the argument on the importance of shared narratives and collective action in transforming sociotechnical systems.
- Dotson (2017): This reference was used in the Community and Commons Concept section to clarify the spectrum of community engagement in the data commons process.
- Morozov (2015) and Fisher (2009): These references were added in the Manifesto Content and Narrative section to discuss the difficulty of imagining non-capitalist futures in the context of data governance and how the manifesto seeks to explore such alternatives.
- Purtova & van Maanen (2023): This reference was integrated into the conclusion to emphasize the need for practical alignment between governance models and the challenges posed by data capitalism.

We believe the revisions made based on your feedback have strengthened the manuscript by providing more clarity on the theory of change, the role of participants, and the manifesto’s impact. The integration of the recommended references has also enriched the theoretical framework, ensuring that the paper is well-grounded in current academic debates on data governance and commons. We look forward to your further comments and hope that the revisions meet your expectations.

# Reviewer report: Reviewer response for version 1

**DOI:** 10.21956/openreseurope.18393.r39845

**Published:** 2024-05-13

**Authors:** Beckett Sterner

**Affiliations:** 1 Arizona State University, Tempe, Arizona, USA

The article discusses the process and results of a pilot effort, Digital Democracy and Data Commons, as part of the EU Decentralized Citizen Owned Data Ecosystems project. The article focuses on a six-month effort to write a collaborative manifesto on digital democracy and data commons. The authors situate the effort in broader scholarship on manifestos as a type of genre and co-creation as a methodology. The authors also present some analysis of how the manifesto developed across versions, including relative changes in emphasis on data compared to democracy and commons.

 An overall comment is that the manuscript seems to fall in between an open letter and a research article. According to the submission instructions, "Open letters are short, peer-reviewed articles discussing policies relevant to a broad research community, presenting guidelines or white papers, or announcing new initiatives. An Open Letter should usually represent the views of a Horizon 2020 and/or Horizon Europe project or group of projects; publication does not imply endorsement by the European Commission.

 An Open Letter should not include new data and analysis." I think this applies well to the manifesto itself, but the manuscript focuses more on describing and analyzing the process of creating the manifesto. I would suggest that the authors make a decision about whether the primary focus is the content and importance of the manifesto itself (open letter option) or the process of writing the manifesto (research article option).

 1. More context about the DECODE project and the Decidim software would be helpful. The current text does not provide enough concrete specifics about who the immediate audience of the manifesto is supposed to be, why the project sought to develop a manifesto in particular, and the significance of DECODE adopting the manifesto. For example, the last paragraph of the "data commons manifesto within the DDDC pilot" subsection doesn't help the reader understand how the manifesto exactly connects to the technological, narrative, and social dimensions. This would also help motivate why the project believed it needed a new manifesto as opposed to adopting one of the several others that are cited in the text.

 2. In participatory/co-creation research, it is essential to characterize who was invited to participate (what was the general population being recruited? How were they recruited?), who actually contributed, and how the project handled issues of power and inequality. Right now the manuscript only addresses those questions too late as limitations mentioned in the discussion. The authors should provide more specifics about the demographics and prior backgrounds/experiences of participants in the "Co-creation approach" section. The authors should clarify their position in the manifesto process along with any other project leadership. I noticed that the manifesto does not list authors online. How was the decision about authorship of this article made?

 3. The quantitative analysis of draft versions is interesting, but it would be more useful to understand the major points of disagreement that happened during the writing process and how they were resolved or set aside. Also, were there key points that were taken for granted based on the prior beliefs and values of the participants? A closer analysis of the text of the manifesto drafts (or of discussion during the writing process) would be valuable for a research article format.

Where applicable, are recommendations and next steps explained clearly for others to follow? (Please consider whether others in the research community would be able to implement guidelines or recommendations and/or constructively engage in the debate)

Partly

Does the article adequately reference differing views and opinions?

Yes

Are all factual statements correct, and are statements and arguments made adequately supported by citations?

Yes

Is the rationale for the Open Letter provided in sufficient detail? (Please consider whether existing challenges in the field are outlined clearly and whether the purpose of the letter is explained)

Yes

Is the Open Letter written in accessible language? (Please consider whether all subject-specific terms, concepts and abbreviations are explained)

Yes

Reviewer Expertise:

Philosophy, Science and Technology Studies, Knowledge Commons

I confirm that I have read this submission and believe that I have an appropriate level of expertise to state that I do not consider it to be of an acceptable scientific standard, for reasons outlined above.
